# Susceptibility of pruning wounds to grapevine trunk diseases: A quantitative analysis of literature data

**DOI:** 10.3389/fpls.2023.1063932

**Published:** 2023-02-23

**Authors:** Maria Chiara Rosace, Sara Elisabetta Legler, Irene Salotti, Vittorio Rossi

**Affiliations:** ^1^ Department of Sustainable Crop Production, Università Cattolica del Sacro Cuore, Piacenza, Italy; ^2^ HORTA S.r.l., Piacenza, Italy

**Keywords:** artificial inoculation, Botryosphaeria dieback, Esca complex, Eutypa dieback, fungal pathogens

## Abstract

**Introduction:**

Pruning wounds are the main entry points for fungi causing grapevine trunk diseases (GTDs). Several studies identified factors influencing the temporal dynamics of wound susceptibility, which include the fungal species and inoculum dose, weather conditions, grape variety, pruning date, and so forth. Here, we conducted a quantitative analysis of literature data to synthesise outcomes across studies and to identify the factors that most affect the length of pruning wound susceptibility.

**Methods:**

We extracted data on the frequency at which the inoculated wounds showed GTD symptoms or an inoculated pathogen was reisolated following artificial inoculation at the time of pruning or in the following days. A negative exponential model was fit to these data to describe changes in wound susceptibility as a function of time since pruning, in which the rate parameter changed depending on specific factors.

**Results and Discussion:**

The results show that wound susceptibility is high at the time of pruning, and they remain susceptible to invasion by GTD fungi for months after pruning. Infection incidence on wounds was higher for fungi associated with Botryosphaeria dieback than those associated with Eutypa dieback or Esca complex, and wound susceptibility decreased faster for Eutypa dieback than for other GTD agents. Grapevine variety and pruning season also affected the wound susceptibility period. Sauvignon Blanc remains susceptible to GTDs longer than other varieties. We also found that the time of pruning can affect infection dynamics, especially for more susceptible varieties. The results increase our understanding of GTD epidemiology and should help growers control infections.

## 1 Introduction

Grapevine trunk diseases (GTDs) cause serious economic losses to the grape industry worldwide (Fontaine et al., 2016b; Mondello et al., 2018a). The casual agents include a wide range of taxonomically distant fungi (Gramaje et al., 2018; Mondello et al., 2018b) that can affect the plant alone or together. In addition to causing external symptoms on foliage and clusters, these pathogens can cause internal wood discoloration. An unpredictable discontinuity in the expression of symptoms is a characteristic of these diseases (Mugnai et al., 1999). GTDs include a range of diseases that affect adult and young vines. Esca complex, Botryosphaeria dieback, and Eutypa dieback are considered major GTDs of adult vines (Claverie et al., 2020).

The Esca complex has been associated with a number of phylogenetically diverse fungi (Mugnai et al., 1999), including both Ascomycota and Basidiomycota. Esca-associated ascomycetes include the vascular pathogens *Phaeomoniella chlamydospora* and *Phaeoacremonium minimum* (syn. *Pm. aleophilum*) (Úrbez-Torres et al., 2014), and other species of *Phaeoacremonium*. Wood-decay basidiomycetes include *Fomitiporia mediterranea* in Europe (Moretti et al., 2021), and other pathogens belonging to the genera *Fomitiporella, Fomitiporia, Inocutis, Inonotus, Stereum*, and *Phellinus* in non-European countries (Cloete et al., 2011; White et al., 2011); these fungi have been isolated from infected vine trunks, but their role in the disease aetiology has not been completely understood (Surico et al., 2006; Bertsch et al., 2013; Gramaje et al., 2018), and is being reconsidered in recent years.

Botryosphaeria dieback is caused by more than 20 species in the Botryosphaeriaceae family, including *Botryosphaeria dothidea*, *Lasiodiplodia theobromae*, *Dothoriella viticola*, *Neofusicoccum parvum*, *N. australe*, *N. luteum*, *N. ribis*, *Diplodia seriata*, and *D. mutila* (Van Niekerk et al., 2004; Taylor et al., 2005; Úrbez-Torres and Gubler, 2009; Amponsah et al., 2012b; Bertsch et al., 2013). Eutypa dieback is caused by *Eutypa lata* and other Diatrypaceae species (Trouillas and Gubler, 2010; Luque et al., 2012). These pathogens can be recovered from the affected wood alone or in combination with other fungi, such as *Pa. chlamydospora*, *Pm. aleophilum*, *Sphaeropsis malorum*, and *Diaporthe ampelina* (Péros et al., 1999).

GTD symptoms are multifaced and include dieback of spurs and arms, discoloration or internal streaking of wood, sectorial wood necrosis and white rot; since plants can be affected by multiple fungi at the same time, some symptoms can overlap among GTDs (Gramaje et al., 2018). Wood discoloration and decay result from a number of structural and physiological changes caused by cellulolytic and ligninolytic enzymes produced by the fungi, vascular occlusion due to gels and gums secreted by the affected xylem parenchyma cells, or necrosis of xylem parenchyma cells as the result of fungal toxins (Bertsch et al., 2013; Claverie et al., 2020). All of these changes lead to an alteration of xylem vessel functions, which results in reduced water and nutrient movement (Mugnai et al., 1999; Sparapano et al., 2000; Andolfi et al., 2011). A detailed description of symptoms in relation to the different GTDs has been recently reported by (Mondello et al., 2018b). Leaves, from which the GTD fungi have never been isolated (Bertsch et al., 2013), also show a variety of symptoms, which have been also described (Mugnai et al., 1999; Amborabé et al., 2001; Mondello et al., 2018b); wood and xylem vessel alteration, fungal toxins and deposition of secondary metabolites all contribute to the expression of disease symptoms (Tabacchi et al., 2000; Bruno et al., 2007). The epidemiology of GTDs is not fully understood. The fungi are known to spread *via* airborne spores (Billones-baaijens et al., 2017), splashing rain (Larignon and Dubos, 2000), or arthropods (Moyo et al., 2014; Kalvelage et al., 2021), even though details on the spread of inoculum in the vineyard are not wholly explained. Spores germinate, and germ tubes enter vine tissues through any type of wound (Graniti et al., 2000; Makatini, 2014) that exposes the xylem; fungi then colonise the wood tissue by growing in and around xylem vessels and parenchyma cells. Pruning wounds are considered a relevant entry point for GTD fungi (Gramaje et al., 2018), and protection of pruning wounds in the period of susceptibility is considered important for reducing fungal penetration into the wood (Halleen et al., 2010; Di Marco et al., 2022).

A number of studies have investigated the dynamics of pruning wound susceptibility to fungal invasion (Petzoldt et al., 1981; Munkvold and Marois, 1995; Eskalen et al., 2007; Serra et al., 2008; Amponsah, 2010; Úrbez-Torres and Gubler, 2011; Amponsah et al., 2014; Luque et al., 2014; Ayres et al., 2016; Elena and Luque, 2016). All of these studies indicate that pruning wounds are highly susceptible to fungal invasion at the time of pruning and that susceptibility declines over the following weeks. These studies were conducted for ascomycetes but not for basidiomycetes, probably because the role of the latter fungi in GTDs has been reconsidered only recently.

These studies collectively show that the wound susceptibility and its decrease over time are influenced by the fungal species, weather conditions, geographical region, grape variety, trellis system, endophytic bacterial microbiome, pruning date, the total surface area of pruning wounds, and vineyard management practices (Larignon and Dubos, 2000; Gu et al., 2005; Borgo et al., 2008; Úrbez-Torres and Gubler, 2011; Bertsch et al., 2013; Billones-Baaijens et al., 2013a; Andreini et al., 2014; Luque et al., 2014; Shafi, 2016; Lecomte et al., 2018; Martínez-Diz et al., 2020; Bekris et al., 2021; Henderson et al., 2021). Wound susceptibility is also influenced by plant response to pruning (Gubler et al., 2005). When a plant is cut or mechanically damaged, a response takes place at both cellular and tissue levels, which include biochemical changes like phenol accumulation, phytoalexin production, synthesis of hydrolytic enzymes, and cell wall reinforcement with suberin and/or lignin (Mundy and Manning, 2011); for example, high lignin production in cell walls has been reported to reduce susceptibility to *E. lata* (Rolshausen et al., 2008). The wound-inducible polyphenol oxidase (PPO) is also related to plant resistance and the production of the above compounds, as reviewed by Fontaine et al. (2016a). In addition to the biochemical changes, the xylem responds to wounding with gel closures in winter and tyloses in summer (Mundy and Manning, 2011). Because of this complexity, synthesizing the knowledge on the susceptibility of pruning wounds to fungal invasion is difficult.

Claverie et al. (2020) recently conducted a literature analysis and provided qualitative information on factors affecting pruning wound susceptibility to GTD fungi. To date, however, a quantitative analysis of the literature data has not been performed. Quantitative analysis of the results coming from different studies is becoming important to increase the statistical power of single studies and for summarising the available scientific knowledge on a topic (Noordzij et al., 2009). This is also true for plant pathology, where “formal” meta-analysis and other quantitative analyses of literature data are increasingly being used (Madden and Paul, 2011; Ngugi et al., 2011; Philibert et al., 2012; Scherm et al., 2014; Ji et al., 2022).

In this study, we performed a systematic literature search and extracted quantitative data from published papers with the following goals: (i) to quantitatively appraise and synthetise outcomes across studies on the dynamics of wound susceptibility to GTD fungi; (ii) to determine whether wound susceptibility depends on the pruning season, grapevine variety, the pathogen that is involved, or by the interaction of these factors; and (iii) to identify knowledge gaps that require further research.

## 2 Materials and methods

### 2.1 Systematic literature search

**Figure 1:**
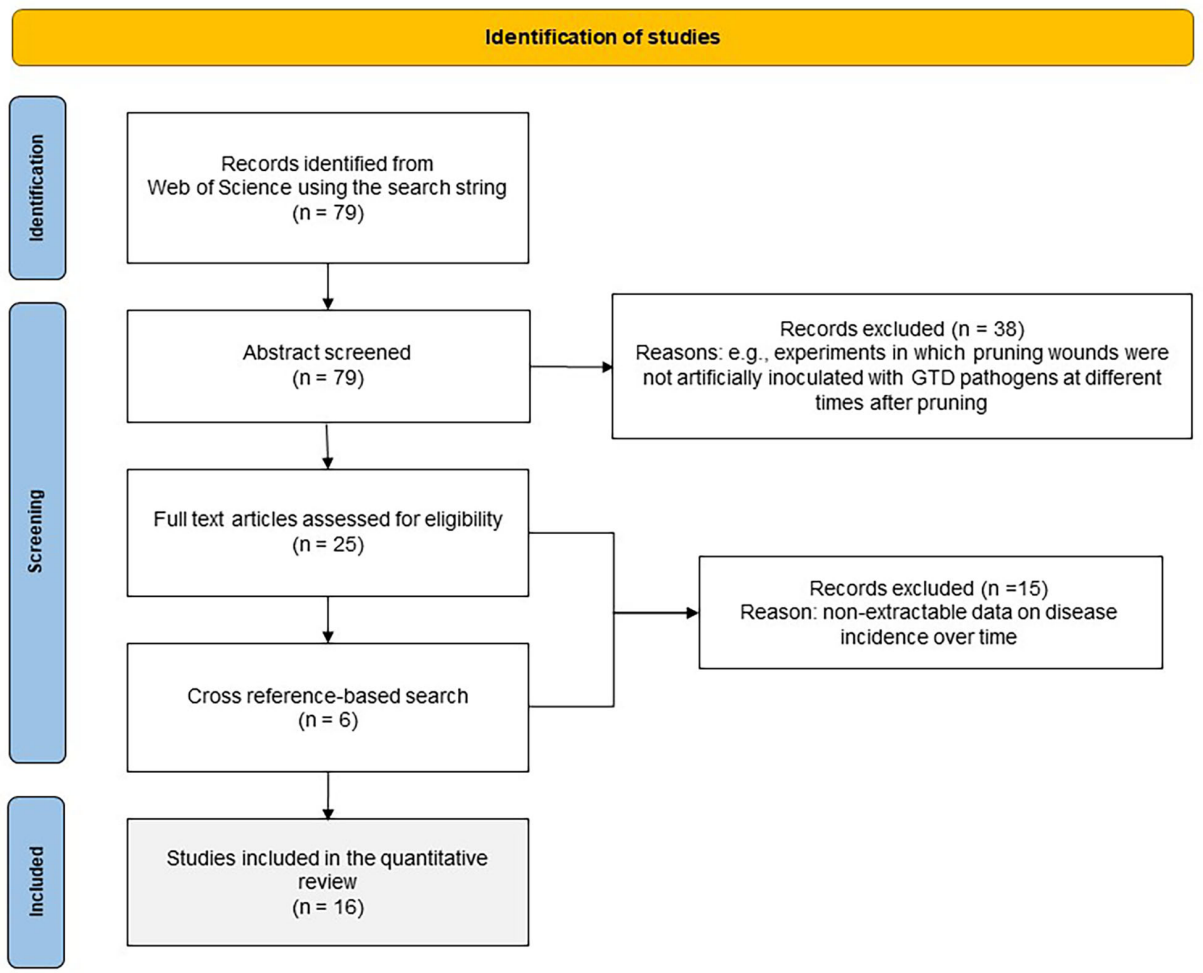
PRISMA flow diagram showing the procedure used for selection of studies for the quantitative analysis.

A systematic literature search was conducted in the Web of Science (WoS) Databases (which include the Web of Science Core Collection, BIOSIS, Derwert, KCI-Korean, Russian Science, SciELO, Data Citation index, and MEDLINE^®^)(Clarivate Analytics, 2020) using the following search string: *Grapevine* AND *Trunk* AND *Disease$* AND *Wound$* AND (*susceptibility* OR *age*). The operator AND indicates that the words must occur simultaneously in the search results, while OR indicates that the selected study must contain any of the terms separated by the operator; the dollar sign ($) represents zero or one character (e.g., “wound” or “wounds”). No restrictions on language or publication date were used. The search detected a total of 79 papers that were exported to EndNote™ online (Clarivate Analytics, online) and that were first screened based on the title and abstract. To be selected, papers had to first satisfy the following criteria: (i) the papers were published in peer-reviewed scientific journals, conference proceedings, scientific reports, or peer-reviewed theses; (ii) they describe experiments in which pruning wounds were artificially inoculated with GTD pathogens at different times after pruning; (iii) they contain quantitative information in the main text, tables, or figures on the percentage of inoculated wounds that were infected (e.g., by observing internal symptoms, or by reisolating the fungal pathogen after artificial inoculation); and (iv) the studies had an experimental design with replicates and were conducted with potted plants or field plants. The papers fulfilling these eligibility criteria were considered to be of potential interest, and the full-text manuscripts were retrieved and reviewed. Additionally, a cross reference-based search of relevant studies was performed to include reports of other studies that might have been eligible for the review and to reduce the risk of missing relevant information due to the choice of search terms. This procedure is shown in **Figure 1** as a PRISMA flow diagram (Koricheva and Gurevitch, 2014).

The cut-off date for publications to be considered for inclusion in this quantitative analysis was 25 July 2022.

### 2.2 Data extraction

Data on the percentage of pruning wounds that showed internal disease symptoms or from which the pathogen was re-isolated following artificial inoculation (hereafter referred to as “disease incidence”) at different times after pruning were extracted from the original papers. All the inoculations were done by depositing a spore suspension (concentration provided as conidia/mL, or ascospores/spores per wound) on the pruning wound surface. Additional details can be found in **Supplementary Material S3**.

Data in the main text or tables were extracted directly, while data in graphs were extracted with WebPlotDigitalizer version 4.3 (Rohatgi, 2020), an online tool that supports the extraction of numerical data from different types of graphs in a PNG or JPEG format. Unfortunately, it was not possible to extract data concerning the within-study variance because only five original articles contained information on the residual error component and the number of replicates (González-Domínguez et al., 2018). To retrieve missing information, we contacted the corresponding authors of the papers were contacted *via* email, but we received only a few replies, in which the authors declared they are not able to provide us with the original data or missing information, with only one exception.

Extracted data were organised in a database containing the selected studies (i.e., articles), cases within a study (i.e., single location or year or variety inoculated with a single pathogen), age of the pruning wounds (the time between pruning and pathogen inoculation, as defined before), and the disease incidence (as defined before). The time at which the pathogens were artificially inoculated on pruning woods was expressed as days after pruning (DAP); the day of pruning was considered as time zero (t_0_). For instance, in an experiment in which pruning wounds were inoculated with a pathogen immediately after pruning and then 7, 14, 21, and 28 days later, the times were referred to as t_0_, t_7_, t_14_, t_21_, and t_28_.

Relevant information on each case was also included in the database in order to extract subsets of data for considering the following main factors that could potentially affect the relationship between disease incidence and DAP were the pruning period (season), identity of the GTD, identity of the inoculated pathogen, and grapevine variety. The pruning period included three “categories”: early-season pruning (i.e., November for the Northern Hemisphere, no data for the Southern Hemisphere were found), mid-season pruning, (i.e., December and January for the Northern Hemisphere and June for the Southern Hemisphere), and late-season pruning (i.e., February and March for the Northern Hemisphere and July and August for the Southern Hemisphere). Identity of GTD included three categories: the Esca complex (EC, cases in which pruning wounds were inoculated with *Pm. minimum* or *Pa. chlamydospora*), Botryosphaeria dieback (BD, cases in which pruning wounds were inoculated with *D. seriata*, *L. theobromae*, *N. parvum*, or *N. luteum*), and Eutypa dieback (ED, cases in which pruning wounds were inoculated with *E. lata* (previously named *E. armeniacae*). There are eight identities for the inoculated pathogens: *Pa. chlamydospora*, *Pm. minimum*, *D. seriata*, *L. theobromae*, *N. parvum*, *N. luteum* and *E. lata*. Ten grapevine varieties: Cabernet Sauvignon, Chardonnay, Chenin Blanc, Merlot, Sauvignon Blanc, Thompson Seedless, Grenache, Pinot Noir, Shiraz, or Tempranillo.

### 2.3 Data analysis

Since it was not possible to extract data on the within-study variance of the different studies, it was impossible to fit a multivariate random effects model *via* linear (mixed-effects) models, and perform a formal meta-analysis. Therefore, we made a quantitative analysis of pooled data coming from the different studies considering the among-study variability only. The weaknesses of this approach are discussed in the next paragraphs.

We first used boxplots (Williamson et al., 1989) to study the effects of the main factors on the disease incidence at the time of pruning (t_0_). To assess the differences between the main factors at t_0_, we used generalised linear models (GLMs). GLMs provide regression analysis and analysis of variance for one dependent variable by one or more factors, allowing the test of the null hypotheses about the effects of other factors on the means of various groupings of a single dependent variable (Neal and Allgar, 2005). Because there was under-dispersion of data (i.e., lower variability than expected by the binomial model), a quasibinomial family distribution was used. The function *glht* (general linear hypotheses) was used for *post hoc* comparisons to compute contrasts between each combination of the factors by using Tukey’s method. The raw data used are available as **Supplementary Material S1**.

To study the temporal dynamics of pruning wound susceptibility, the disease incidence at t_0_ was set at 1, and the disease incidences at the following inoculation times were rescaled to 1 and expressed on a 0 to 1 scale. For instance, if the disease incidence was 85% for wounds inoculated at t_0_, and was 55%, 32%, 10%, and 3% for wounds inoculated at 7, 14, 21, and 28 DAP, then t_0 =_ 1.00, t_7 =_ 0.65 (i.e., 55/85), t_14 =_ 0.38 (i.e., 32/85), t_21 =_ 0.12 (i.e., 10/85), and t_28 =_ 0.04 (i.e., 3/85).

Average (and standard error) of the rescaled disease incidence in the different cases was then calculated for each timing after pruning (DAP) and fit to a negative exponential equation in the following form:


[1] $$Y_{t}=e^{-at}$$

where *Y_t_* is the rescaled disease incidence (dependent variable, from 0 to 1) at time *t*; *t* is the time of fungal inoculation after pruning (independent variable, in days: DAP); parameter *a* is the rate at which *Y* decreases as *t* increases, which was estimated for all data, main factors, and interactions (for a total of 71 estimates) by using nonlinear regression procedures (*nls*); standard errors of the parameter *a* given by the *nls* function were used to calculate the confidence interval of the predicted Y values.

Goodness-of-fit of equations [1] were calculated as follows: NSE (Nash-Sutcliffe efficiency), i.e., the ratio of the mean square error to the variance in the observed data, subtracted from unity (when the error is zero, NSE = 1, and the equation provides a perfect fit); W index of agreement, i.e., the ratio between mean square error to total potential error (W = 1 represents a perfect fit); root mean square error (RMSE), i.e., the fit standard error of the regression, which is calculated as the square root of the mean square error (RMSE represents the average distance of real data from the fitted line); coefficient of residual mass (CRM), which is a measure the tendency of the model to over- or underestimate the observed data (a negative CRM indicates a tendency of the model toward overestimation); R^2^, which represents the proportion of experimental variability explained by the selected model (R^2^ = 1 represents a perfect fit); and the concordance correlation coefficient (CCC), which is calculated as a measure of model accuracy (the CCC is the product of the Pearson product-moment correlation coefficient between observed and predicted values, and the coefficient C_b_ indicates the difference between the best-fitting line and the perfect agreement line; CCC ranges from -1 to 1, with perfect agreement at 1 (Nash and Sutcliffe, 1970; Loague and Green, 1991; Madden et al., 2007).

To determine whether the temporal dynamics of pruning wound susceptibility were significantly affected by the categories of the main factors and their two-way interactions (three-way interactions were not considered because the number of studies would have been too low), the following null hypothesis was tested: the slope *a* of equation [1] estimated for a category of a main factor (e.g., category EC for the main factor GTD) was not significantly different from that of another category of the same factor (e.g., EC vs. BD). The latter null hypothesis was tested as follows. Equation [1] was expressed in its linear form through data transformation through the natural logarithm function (*ln*), and the intercept of the linearised equation was set at ln(1 + 1) shifting the y-values by ln(2). Significant differences between slopes of the linearised, shifted functions were finally tested using the *lsmeans* package; slopes were extracted using the function *lstrends* and then compared through the function *pairs*, which returns a matrix consisting of *t*-tests for each comparison. There were 529 comparisons in total, and these are shown in **Supplementary Material S2**.

All statistical analyses were conducted with R studio Version 1.4.1717 (RStudio Team, 2021).

## 3 Results

### 3.1 Literature search and database overview

The literature search provided 79 studies, of which 41 were selected for full text analysis. The others were disregarded because they were not considered relevant to this study as they failed to meet the above-mentioned criteria. Two additional papers were selected *via* cross-referencing. After full text analysis, 16 papers were used for quantitative data extraction. The selected papers were published between 1980 and 2022, and were conducted in Australia, New Zealand, California, the USA, South Africa, Italy, Spain, and France; most were performed in the Northern Hemisphere. The key information of these 16 studies is summarised in **Table 1**; further information is available as **Supplementary Material S3**. Since each study considered multiple fungi, years, or vineyards, there were 154 cases in our dataset, for a total of 1110 single data values for disease incidence.

**Table 1:** Main characteristics of the studies selected for the analysis (further information is available in **Supplementary Material – S3**).

*Fungal species*	*Pruning months*	*Years*	*Scale*	*Inoculation time* *(DAP, days after pruning)*	*Country or Region*	*Reference*
*Pm. minimum* *Pa. chlamydospora*	February	2000 2001	Field	0, 30, 60, 90, 120, 150, 180	California	Eskalen et al., 2007
*Pm. minimum* *Pa. chlamydospora* *D. seriata*	January to March	2005 to 2007	Field	0, 7, 14, 21, 28, 35, 42, 49, 56, 63, 70, 77, 84, 91, 98, 105	Italy	Serra et al., 2008
*L. theobromae* *N. parvum*	November to January	2007 2008	Field	0, 12, 24, 36, 48, 60, 72, 84	California	Úrbez-Torres and Gubler, 2011
*D. seriata* *Pa. chlamydospora*	November February	2012 2013	Pots	1, 7, 14, 28, 56, 84	Spain	Elena and Luque, 2016
*Pm. minimum* *Pa. chlamydospora*	December to March	1996 to 1999	Field	0, 7, 14, 21, 28, 35, 42, 49, 56, 63, 70, 77, 84, 91, 98, 105, 112, 119, 126, 133	France	Larignon and Dubos, 2000
*E. lata*	December to February	1991to 1994	Field	0, 7, 14, 21, 28, 35, 42, 49	France	Chapuis et al., 1998
*E. lata*	November to March	1989 to 1991	Field	1, 7, 14, 20 and 28	California	Munkvold and Marois, 1995
*N. luteum* *N. parvum*	February	2014	Pots	0, 7, 14, 28, 42, 56,70	New Zealand	Shafi, 2016
*E. lata*	December to March	1978 1979	Field	0, 7, 14, 21	California	Petzoldt et al., 1981
*E. lata* *Pa. chlamydospora* *N. australe*	July and August	2004 2005	Field	0, 1, 2, 3, 7, 10, 14, 17,21	South Africa	Van Niekerk et al., 2011
*E. lata* *D. seriata* *N. luteum*	June to August	2013 to 2015	Field	1, 7, 14, 28, 42, 56, 84, 112	Australia	Ayres et al., 2016
*E. lata*	August	2000 to 2002	Field	0, 1, 14	Australia	Sosnowski et al., 2008
*E. lata*	February	1977	Field	0, 8, 14, 22	California	Moller and Kasimatis, 1980
*N. luteum*	October-December	2008	Pots	0, 1, 2, 7, 14, 30	New Zealand	Amponsah, 2010; Amponsah et al., 2014
*L. theobromae* *E. lata* *P. minimum* *Pa. chlamydospora*	December	2008-2009	Field	0, 7, 14, 21	California	Herche, 2009
*E. lata* *N. luteum*	July	2013 to 2015	Field	1, 6, 14	Australia	Ayres et al., 2022

In the selected studies, wounds were inoculated at various times after pruning with a maximum of 10 months (Eskalen et al., 2007) on different grapevine varieties and in experiments conducted either in the field or with potted plants located outdoors, for 1 to 4 consecutive years. Three major GTDs were considered, including six studies for the Esca complex (in which the artificial inoculation was conducted with *Pm. minimum* or *Pa. chlamydospora*, nine for Botryosphaeria dieback (with *D. seriata*, *L. theobromae*, *N. parvum*, or *N. luteum*), and nine for Eutypa dieback (with *E. lata*) (**Table 1**). Pruning dates varied between November and March for the Northern Hemisphere, and between June and August for the Southern Hemisphere. In only two studies (Amponsah et al., 2014; Shafi, 2016), vines were pruned during the vegetative period; the data from these two studies were used in the whole dataset but not in the analysis of the pruning period.

### 3.2 Susceptibility of wounds at the time of pruning

**Figure 2:**
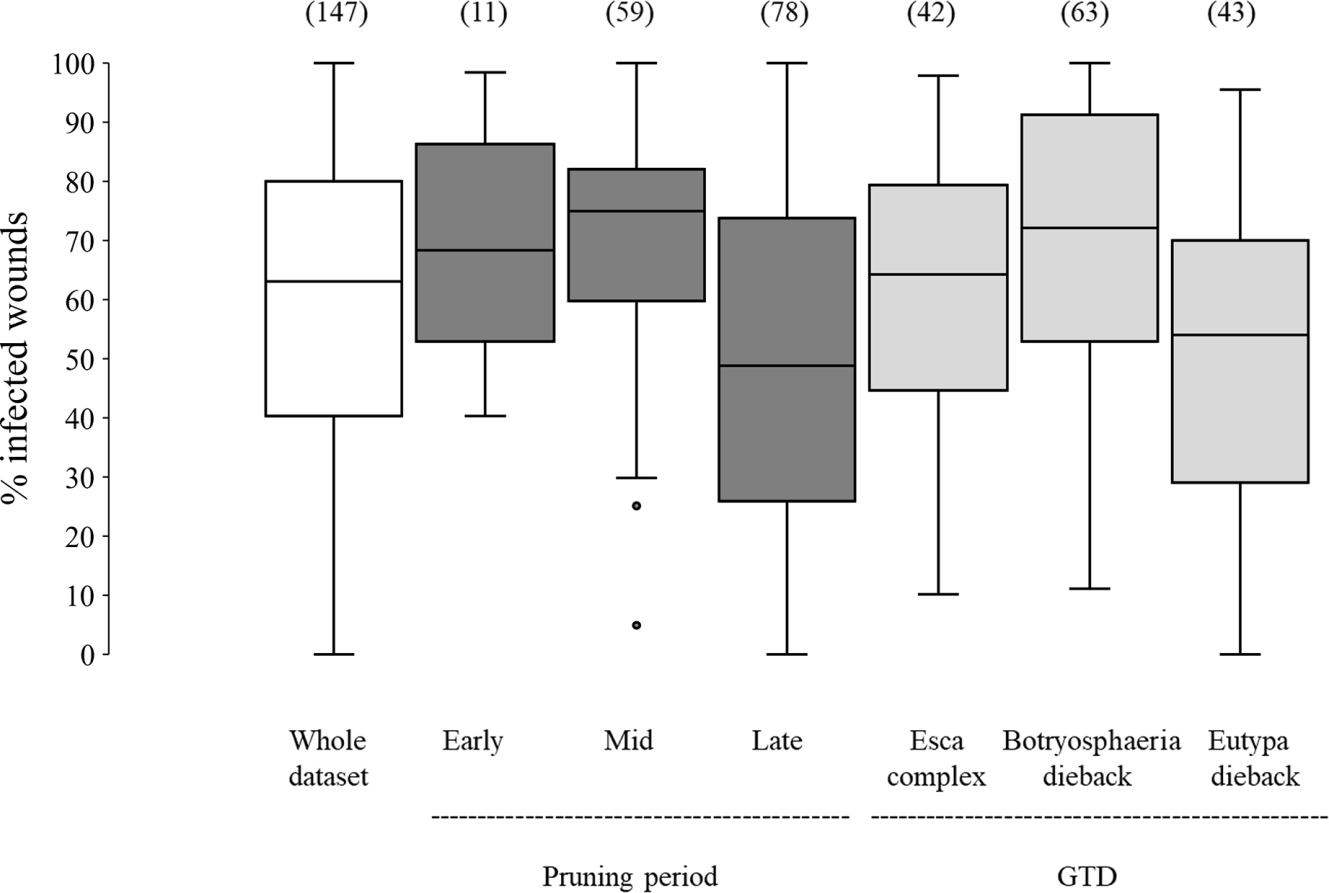
Susceptibility of the pruning wounds at the time of pruning (t_0_) (expressed as the % of the wounds that became infected after being artificially inoculated with fungi associated with grape trunk diseases [GTDs] for the whole dataset (the white box), and for subsets of data concerning pruning periods (the dark-grey boxes) and GTDs (the light-grey boxes). The values in brackets indicate the number of observations available for each subset. Whiskers indicate the lowest and highest values (excluding outliers, represented by black dots); 25% of the data fall below the lower quartile value, and 75% of the data fall below the upper quartile value. The median marks the mid-point of the data and is shown by the line that divides the box into two parts.

**Figure 3:**
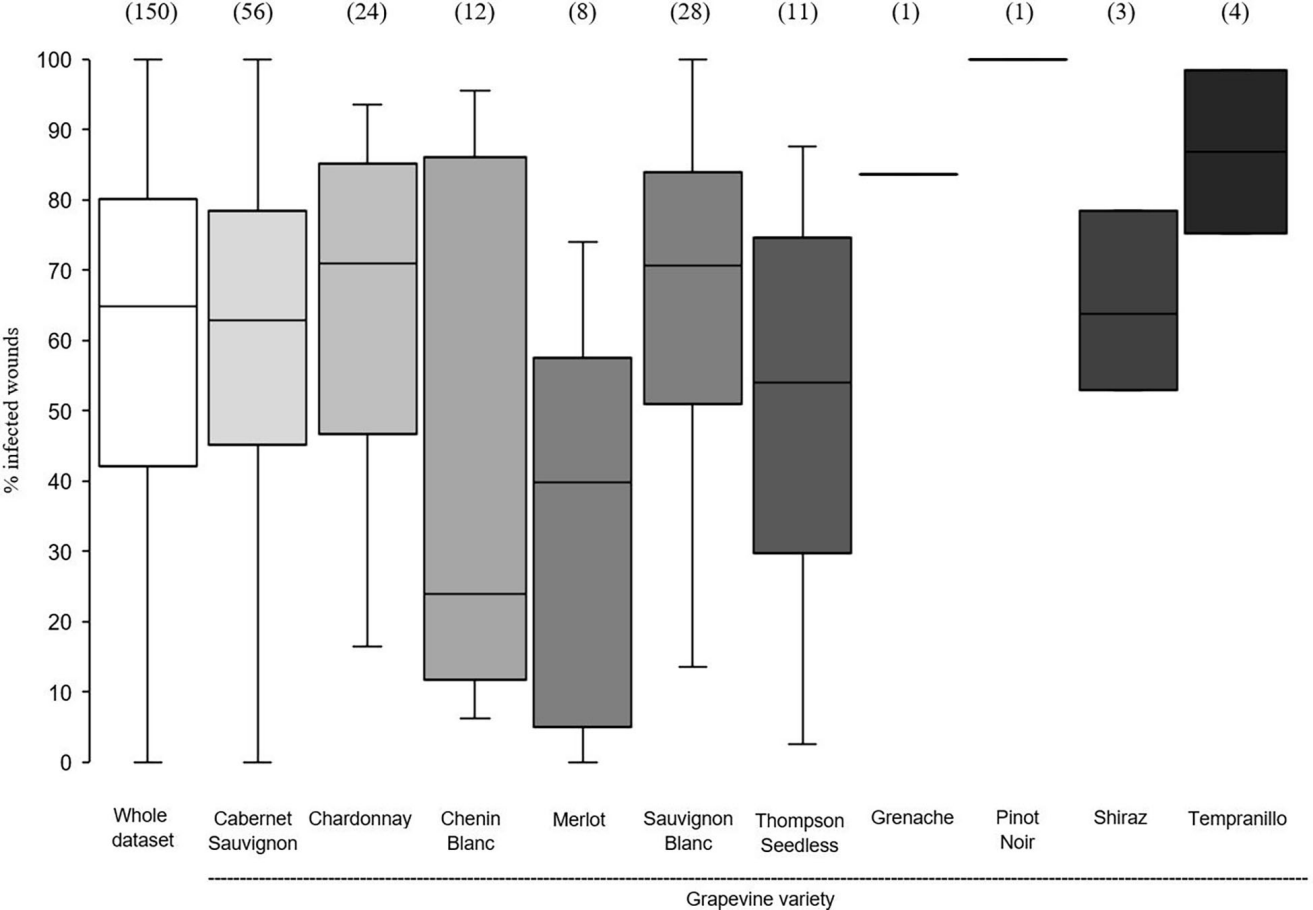
Susceptibility of the pruning wounds at the time of pruning (t_0_) (expressed as the % of the wounds that became infected after being artificially inoculated with fungi associated with grape trunk diseases [GTDs] for the whole data set (the white box) and for subsets of data concerning grapevine varieties. The values in brackets indicate the number of observations available for each subset. Whiskers indicate the lowest and highest values (excluding outliers, represented by black dots); 25% of the data fall below the lower quartile value, and 75% of the data fall below the upper quartile value. The median marks the mid-point of the data and is shown by the line that divides the box into two parts.

In the whole dataset, the median value of disease incidence (wounds being infected or showing GTDs symptoms following artificial inoculation with GTDs fungi) at the time of pruning (t_0_) was 64.0%; the variability, however, was very high with extremes ranging from 0 to 100%, and with 50% of the data ranging from 40.3 to 80.0% (**Figure 2**). When the whole dataset was split based on the pruning period, the disease incidence for wounds inoculated at t_0_ was significantly higher for wounds inoculated at mid-pruning than at late pruning (*P*< 0.001), with early pruning showing intermediate values (**Figure 2**). When the whole dataset was split based on the GTD, the incidence of successful infections was significantly lower for Eutypa dieback than for Botryosphaeria dieback (*P* = 0.032), with intermediate values for Esca complex agents. When the whole dataset was split based on grape variety (**Figure 3**), disease incidence was lower for Chenin Blanc and Merlot than for other varieties; for Chenin Blanc and Merlot,< 40% of inoculated wounds became infected (**Figure 3**). Because of high variability among experiments and fewer data availability for Grenache, Pinot noir, Tempranillo, and Shiraz, no significant differences were found in the pairwise comparisons between these varieties (*P* > 0.058).

### 3.3 Changes in wound susceptibility over time

**Figure 4:**
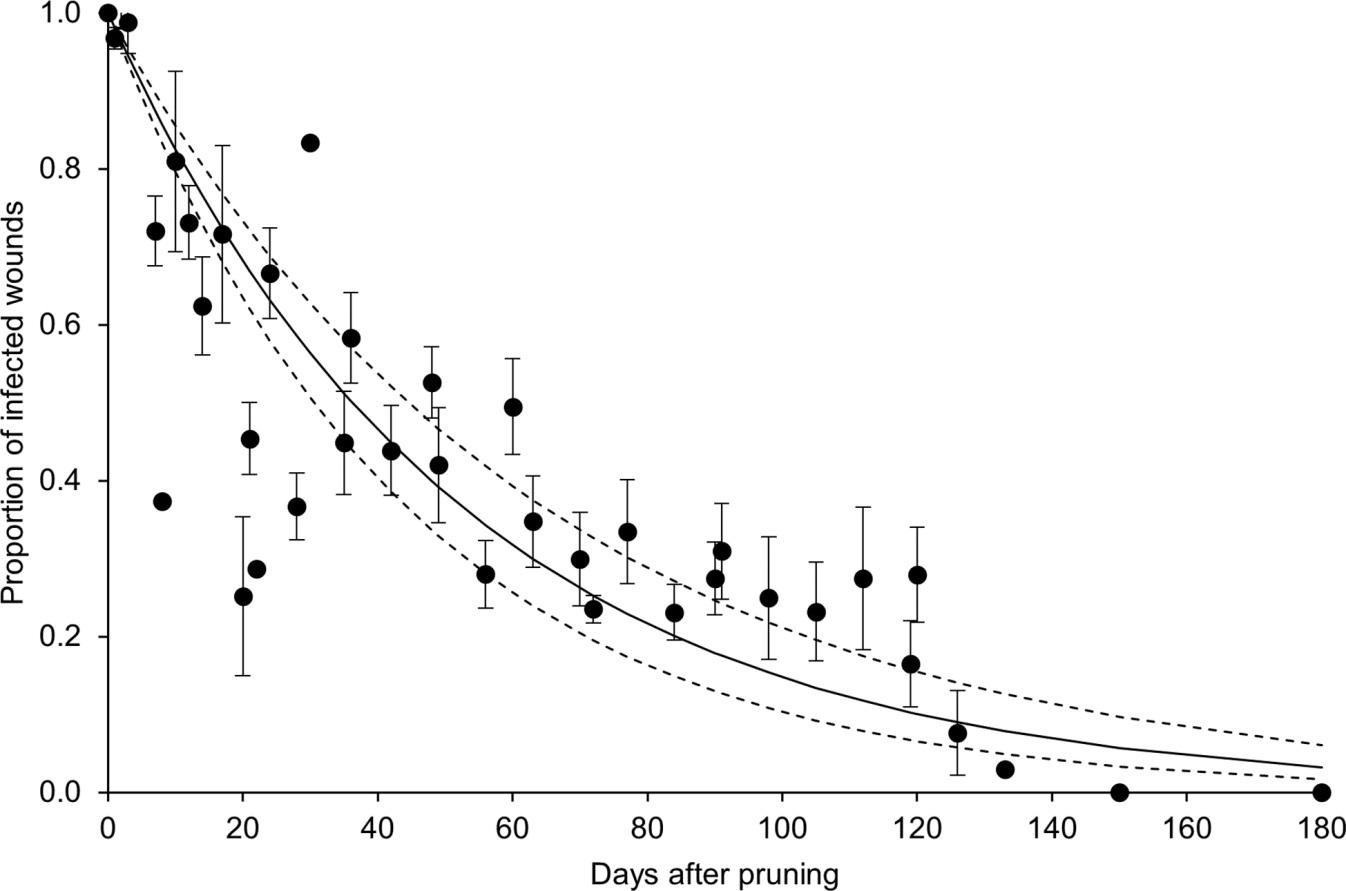
Proportion of pruning wounds that became infected as a function of DAP (number of days after pruning when the wounds were inoculated with fungi associated with grape trunk diseases [GTDs]) for the whole dataset shown in **Table 1**. Dots are averages of the observed values for each day, and whiskers represent the standard error. The full line shows the fit of the observed data with equation [1] (see text and **Table 2**), and the dashed lines show the upper and lower intervals of the distribution (i.e., *a* ± standard error).

In the whole dataset, the disease incidence declined over time after pruning (DAP) when the GTD fungi were inoculated (**Figure 4**). Equation [1] fit the data with *a* = 0.019 ± 0.002, R^2^ = 0.74, CCC = 0.85, NSE = 0.68, W = 0.92, RMSE = 0.16, and CRM = -0.031. The analysis of data distribution with respect to the fit and its confidence interval showed that > 50% of the observed data overlapped with the predicted interval, with some overestimation between 20 and 30 DAP, and underestimation between 90 and 120 DAP (**Figure 4**).

When the whole dataset was divided according to the main factors (pruning period, GTD, grape variety, and fungal species) and their interactions, for a total of 70 combinations, equation [1] fit the observed data rather well with the following ranges of values: R^2^ (0.25 to 1); NSE (0.1 to 0.99); W (0.46 to 1); RMSE (0.02 to 0.72); CRM (-0.08 to 0.12); and CCC (0.3 to 1) (**Tables 2**–**5**).

**Table 2:** Parameters and statistics of goodness-of-fit for equation [1] (see text), which predicts pruning wound susceptibility as a function of time after pruning when wounds were inoculated, for the whole dataset obtained from the studies in **Table 1**, and for subsets of data concerning different categories of the following main factors: pruning period, disease, species inoculated to wounds, and grape variety.

Datasets	n	Parameter estimate		P^1^	Goodness-of-fit					
		*a*	SE		R^2^	NSE	W	RMSE	CRM	CCC
Whole dataset	39	0.019	0.002	NA	0.736	0.682	0.925	0.158	-0.031	0.854
Early pruning	14	0.018	0.003	0.243	0.703	0.703	0.908	0.163	-0.010	0.825
Mid pruning	28	0.020	0.002	0.859	0.697	0.602	0.910	0.154	-0.020	0.827
Late pruning	32	0.021	0.003	0.999	0.50	0.417	0.844	0.23	-0.03	0.710
Esca complex	31	0.016	0.002	0.985	0.796	0.792	0.942	0.149	-0.024	0.890
Botryosphaeria dieback	30	0.015	0.002	0.995	0.666	0.587	0.900	0.157	-0.023	0.812
Eutypa dieback	17	0.049	0.006	*<0.001*	0.846	0.843	0.954	0.138	0.023	0.911
*Phaeomoniella chlamydospora*	31	0.016	0.001	0.999	0.835	0.832	0.955	0.130	-0.022	0.912
*Phaeoacremonium minimum*	26	0.016	0.002	0.999	0.670	0.666	0.896	0.183	-0.030	0.810
*Diplodia seriata*	19	0.011	0.001	0.633	0.616	0.552	0.885	0.152	-0.028	0.783
*Lasiodiplodia theobromae*	12	0.020	0.004	0.752	0.588	0.543	0.875	0.191	-0.036	0.765
*Neofusicoccum parvum*	9	0.023	0.004	0.418	0.727	0.695	0.923	0.142	-0.007	0.852
*Neofusicoccum luteum*	12	0.083	0.008	*0.003*	0.983	0.981	0.995	0.055	0.019	0.990
*Eutypa lata*	17	0.049	0.006	*<0.001*	0.846	0.843	0.954	0.138	0.023	0.911
Cabernet Sauvignon	32	0.024	0.003	0.966	0.642	0.629	0.890	0.189	-0.011	0.798
Chardonnay	12	0.024	0.005	0.753	0.598	0.540	0.878	0.197	-0.019	0.773
Chenin Blanc	10	0.026	0.004	0.591	0.806	0.788	0.930	0.103	0.016	0.869
Grenache	8	0.091	0.018	*0.016*	0.917	0.898	0.976	0.096	0.019	0.954
Merlot	4	0.040	0.010	*0.015*	0.554	0.507	0.858	0.205	-0.004	0.744
Pinot noir	6	0.079	0.010	*0.036*	0.965	0.961	0.990	0.066	0.031	0.981
Sauvignon Blanc	6	0.010	0.001	0.651	0.641	0.587	0.895	0.132	-0.019	0.800
Shiraz	6	0.095	0.020	*<0.001*	0.919	0.912	0.977	0.101	0.085	0.955
Tempranillo	18	0.026	0.003	0.794	0.949	0.943	0.986	0.071	-0.014	0.973
Thompson seedless	13	0.022	0.008	0.999	0.474	0.398	0.836	0.276	-0.036	0.687

^1^P value for the difference between estimates of *a* for the subsets and the whole dataset.

**Table 3:** Parameters and statistics of goodness-of-fit for equation [1] (see text), which predicts pruning wound susceptibility as a function of time after pruning when wounds were inoculated for subsets of the data in **Table 1** that concern the interaction between pruning period and grape trunk disease.

Pruning period Grape trunk disease	n	Parameter estimate		P^1^	Goodness-of-fit					
		*a*	SE		R^2^	NSE	W	RMSE	CRM	CCC
Early pruning										
Esca complex	6	0.020	0.003	0.785	0.930	0.929	0.981	0.079	0.071	0.963
Botryosphaeria dieback	13	0.019	0.004	0.336	0.606	0.603	0.871	0.206	-0.022	0.759
Eutypa dieback	5	0.036	0.007	0.084	0.908	0.852	0.950	0.112	0.039	0.905
Mid pruning										
Esca complex	21	0.021	0.002	0.987	0.858	0.821	0.959	0.113	-0.012	0.921
Botryosphaeria dieback	25	0.010	0.002	0.527	0.388	0.365	0.771	0.216	-0.022	0.600
Eutypa dieback	11	0.057	0.008	*<0.001*	0.881	0.871	0.968	0.115	0.035	0.938
Late pruning										
Esca complex	25	0.015	0.002	0.999	0.730	0.723	0.921	0.163	-0.028	0.851
Botryosphaeria dieback	23	0.024	0.003	0.469	0.767	0.728	0.933	0.148	0.012	0.873
Eutypa dieback	17	0.053	0.008	*<0.001*	0.843	0.828	0.945	0.162	0.008	0.894

^1^P value for the difference between estimates of *a* for the subsets and the whole dataset.

**Table 4:** Parameters and statistics of goodness-of-fit for equation [1] (see text), which predicts pruning wound susceptibility as a function of time after pruning when wounds were inoculated for subsets of the data that concern the interaction between grape variety and grape trunk disease.

Grape variety Grape trunk disease	n	Parameter estimate		P^1^	Goodness-of-fit					
		*a*	SE		R^2^	NSE	W	RMSE	CRM	CCC
Cabernet Sauvignon										
Esca complex	27	0.026	0.005	0.995	0.540	0.519	0.848	0.231	-0.002	0.729
Botryosphaeria dieback	15	0.022	0.003	0.987	0.689	0.633	0.910	0.163	-0.021	0.828
Eutypa dieback	9	0.060	0.009	*<0.001*	0.904	0.901	0.975	0.109	-0.016	0.950
Chardonnay										
Esca complex	4	0.073	0.022	*<0.001*	0.912	0.842	0.944	0.155	0.033	0.894
Botryosphaeria dieback	12	0.023	0.005	0.886	0.658	0.613	0.898	0.176	-0.020	0.810
Eutypa dieback	4	0.059	0.013	*0.028*	0.938	0.890	0.964	0.108	0.032	0.931
Chenin Blanc										
Esca complex	9	0.024	0.006	*<0.001*	0.522	0.511	0.834	0.117	-0.012	0.706
Botryosphaeria dieback	9	0.021	0.004	0.999	0.738	0.723	0.904	0.085	0.015	0.826
Eutypa dieback	10	0.030	0.005	0.413	0.830	0.807	0.938	0.107	0.023	0.881
Sauvignon Blanc										
Esca complex	18	0.010	0.001	0.903	0.805	0.793	0.946	0.098	-0.008	0.897
Botryosphaeria dieback	18	0.010	0.002	0.697	0.254	0.098	0.723	0.227	-0.045	0.497
Tempranillo										
Esca complex	6	0.020	0.003	0.987	0.915	0.915	0.977	0.087	-0.015	0.955
Botryosphaeria dieback	6	0.035	0.004	0.580	0.960	0.949	0.988	0.069	0.010	0.976
Thompson seedless										
Esca complex	7	0.011	0.003	0.987	0.862	0.820	0.937	0.170	0.002	0.882
Eutypa dieback	7	0.057	0.008	*0.001*	0.902	0.899	0.972	0.103	0.022	0.945

^1^P value for the difference between estimates of *a* for the subsets and the whole dataset.

**Table 5:** Parameters and statistics of goodness-of-fit for equation [1] (see text), which predicts pruning wound susceptibility as a function of time after pruning when wounds were inoculated for subsets of the data that concern the interaction between grape variety and pruning period.

Grape variety Pruning period	n	Parameter estimate		P^1^	Goodness-of-fit					
		*a*	SE		R^2^	NSE	W	RMSE	CRM	CCC
Cabernet Sauvignon										
Early pruning	8	0.011	0.004	0.999	0.732	0.620	0.832	0.228	0.059	0.710
Mid pruning	27	0.028	0.002	0.564	0.843	0.834	0.958	0.117	0.002	0.918
Late pruning	24	0.036	0.008	0.999	0.396	0.332	0.783	0.277	0.064	0.623
Chardonnay										
Early pruning	8	0.017	0.002	0.999	0.940	0.929	0.979	0.077	0.007	0.959
Mid pruning	12	0.021	0.005	0.999	0.530	0.491	0.851	0.214	-0.026	0.725
Late pruning	4	0.056	0.003	0.234	0.996	0.994	0.999	0.024	0.016	0.997
Chenin Blanc										
Mid pruning	5	0.033	0.009	0.936	0.624	0.609	0.888	0.144	-0.020	0.784
Late pruning	10	0.028	0.006	0.792	0.732	0.703	0.891	0.178	0.022	0.801
Merlot										
Mid pruning	8	0.048	0.015	0.144	0.445	0.237	0.818	0.230	0.019	0.662
Late pruning	7	0.034	0.033	*<0.001*	0.284	0.234	0.464	0.715	0.053	0.302
Sauvignon Blanc										
Mid pruning	18	0.010	0.001	0.932	0.439	0.307	0.820	0.172	-0.040	0.659
Late pruning	14	0.012	0.001	0.999	0.911	0.911	0.976	0.066	0.005	0.953
Shiraz										
Mid pruning	6	0.145	0.036	*<0.001*	0.914	0.903	0.973	0.108	0.121	0.947
Late pruning	6	0.079	0.023	*0.005*	0.837	0.833	0.953	0.147	0.064	0.909
Tempranillo										
Early pruning	6	0.028	0.005	0.966	0.905	0.898	0.975	0.098	-0.019	0.950
Late pruning	6	0.023	0.002	0.999	0.980	0.977	0.994	0.044	-0.010	0.989
Thompson seedless										
Early pruning	5	0.036	0.007	0.745	0.908	0.852	0.950	0.112	0.039	0.905
Mid pruning	7	0.060	0.011	*0.002*	0.901	0.884	0.965	0.127	0.010	0.930
Late pruning	13	0.019	0.008	0.999	0.359	0.278	0.783	0.331	-0.084	0.589

^1^P value for the difference between estimates of *a* for the subsets and the whole dataset.

**Figure 5:**
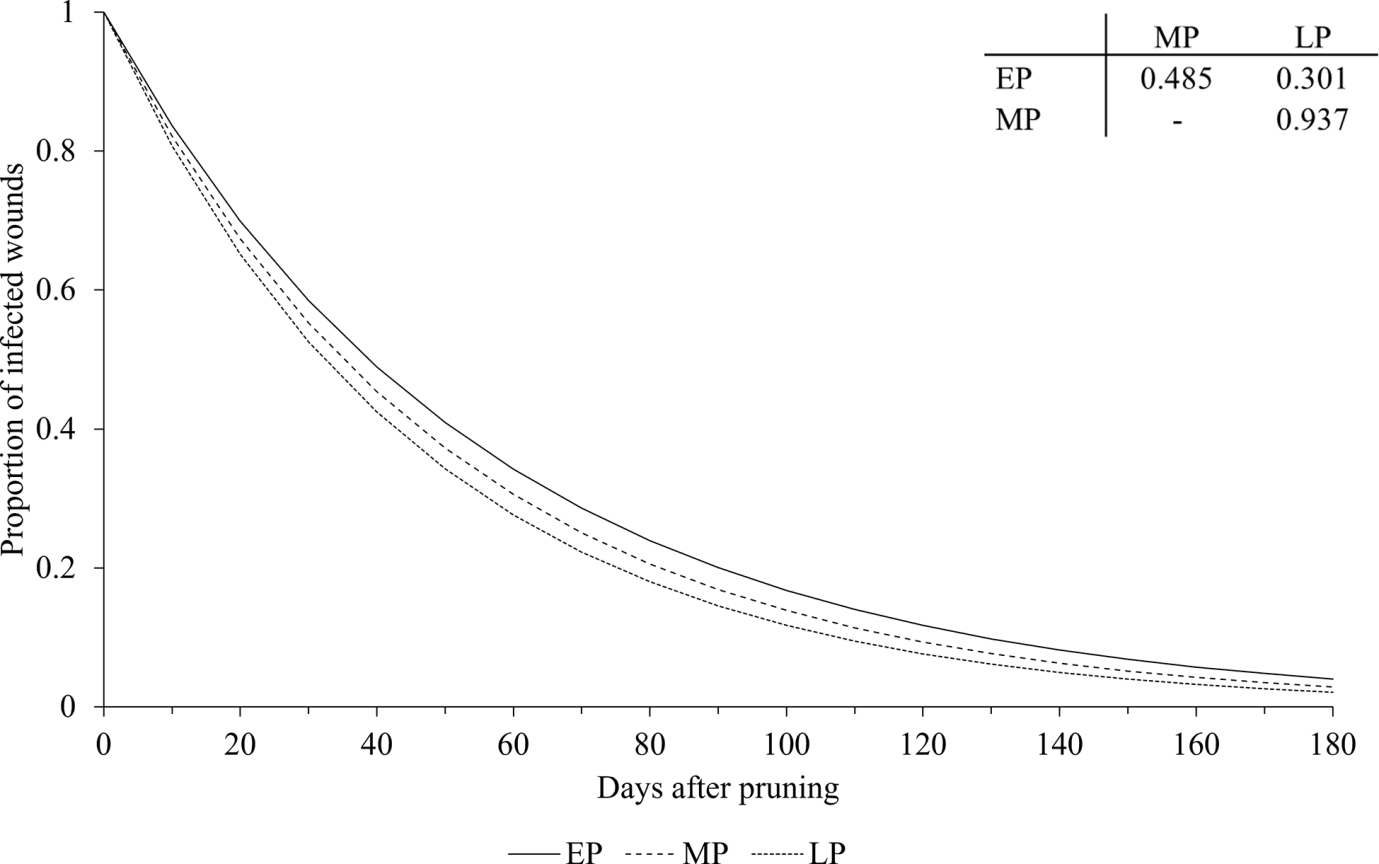
Effect of pruning period on the relationship between the proportion of pruning wounds that became infected (Y axis) and the time after pruning (DAP) when the wounds were artificially inoculated with GTD fungi (X axis). Early pruning period (EP - full line), mid pruning period (MP - dashed line), and late pruning period (LP - dotted line). Lines were predicted by equation [1] (see text). The table shows the P values for the differences between the estimates of parameter *a* in equation [1] (see **Table 2** for estimates of *a* for pruning periods).

**Figure 6:**
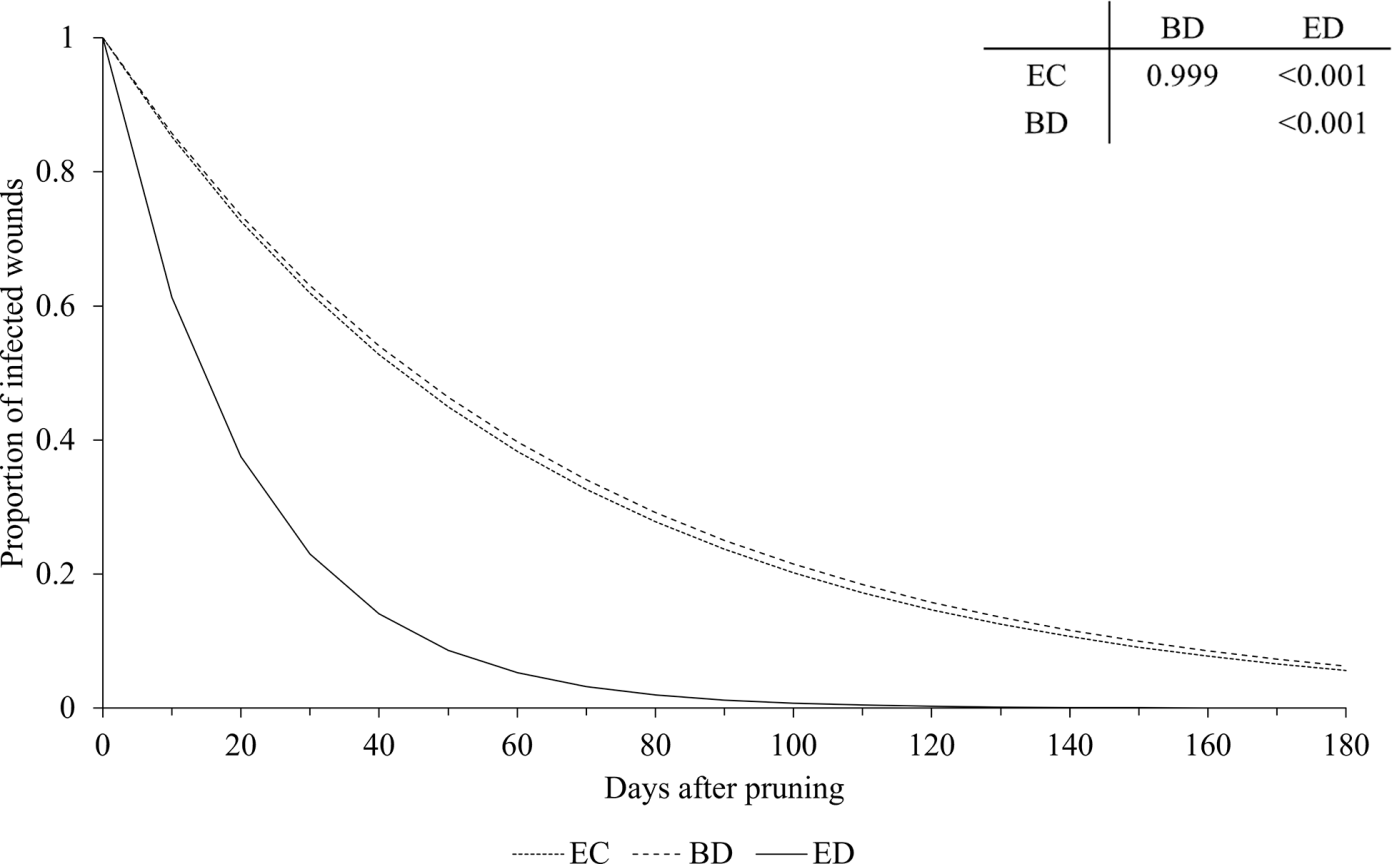
Effect of GTDs on the relationship between the proportion of pruning wounds that became infected (Y axis) and the time after pruning (DAP) when the wounds were artificially inoculated with GTD fungi (X axis). Esca complex (EC - dotted line), Botryosphaeria dieback (BD - dashed line), and Eutypa dieback (ED - full line). Lines were predicted by equation [1] (see text). The table shows the P values for the differences between estimates of parameter *a* of equation [1] (see **Table 2** for estimates of *a* for the GTDs).

The pruning period (as a main factor) was not significantly related to the dynamics of wound susceptibility, because the slope parameters calculated for early (*a* = 0.018), mid (*a* = 0.020), and late (*a* = 0.021) pruning were not significantly different from *a* based on the whole dataset (P > 0.243; **Table 2**) or from each other, with *P* > 0.485 (**Figure 5**). Disease as a main factor, on the contrary, showed a significant effect. Wound susceptibility decreased over a shorter period after pruning for Eutypa dieback (*a* = 0.049) than for the Esca complex (*a* = 0.016) or Botryosphaeria dieback (*a* = 0.015), with *P<* 0.001 (**Table 2** and **Figure 6**). For Botryosphaeria dieback, a significant interaction between pruning period x GTD (**Table 3**) showed that the susceptibility decreased faster for late pruning (*a*=0.024) and early pruning (*a* = 0.019), which were not significantly different from each other (*P* = 0.999), than for mid-pruning (*a* = 0.010; *P* = 0.021 and 0.029, respectively) (**Supplementary Material S2**).

Wound susceptibility decreased faster when pruning wounds were inoculated with *E. lata* (*a* = 0.049) than with other fungal species (*a* ranged from a minimum of 0.011 for *D. seriata* to a maximum of 0.023 for *N. parvum*, **Table 2**; with *P<* 0.001, **Supplementary Material S2**), with the exception of *N. luteum* (*a* = 0.083; *P* = 0.019). The latter species was also different from the two main species involved in the Esca complex, *Pa. chlamydospora* and *Pm. minimum* (*a* = 0.016 for both species; *P* = 0.001 and 0.002, respectively), and from *D. seriata* (*a* = 0.011; *P<* 0.001), which is part of the same GTD (i.e., Botryosphaeria dieback).

Wounds were susceptible for a shorter period for Shiraz, Grenache, Pinot noir, and Merlot (in decreasing order, with *a* ranging from 0.095 to 0.040) than for Cabernet Sauvignon, Chardonnay, Chenin Blanc, Tempranillo, and Thompson seedless (in decreasing order, with *a* ranging from 0.026 to 0.022); the pairwise comparisons between varieties in the first and second of these groups were in most cases significant groups (**Table 2** and **Supplementary Material S2**). Sauvignon Blanc had the longest period of wound susceptibility (*a* = 0.010), and was different from the first group of varieties but not from the second. No significant differences were found for the pruning period within each variety (*P* > 0.593), with the exception of Thompson Seedless, in which the susceptibility of pruning wound declined slower with late pruning (*a* = 0.019) than with early or mid-pruning (*a* = 0.036 and 0.060, respectively; *P* = 0.001) (**Table 3** and **Supplementary Material S2**). Nevertheless, differences were detected between varieties within the same pruning seasons. Wounds of Sauvignon Blanc pruned in mid-season (*a* = 0.01) were susceptible for a longer time than wounds of Cabernet Sauvignon (*a* = 0.028; *P* = 0.02) or Thomson seedless (*a* = 0.06; *P*< 0.001) pruned in the same season. Differences among varieties were not significant with late-season pruning (**Supplementary Material S2**). No data were available for early-pruned Sauvignon Blanc.

Differences were also found for some varieties or pruning seasons among GTDs. For Cabernet Sauvignon, the decrease in wound susceptibility over time after pruning was faster for Eutypa dieback (*a* = 0.060) than for Botryosphaeria dieback or the Esca complex (*a* = 0.022 and 0.026, respectively; *P<* 0.001) (**Table 4** and **Supplementary Material S2**). Similarly, decline in wound susceptibility for Thompson seedless was faster for Eutypa dieback (*a* = 0.057) than for Esca complex (*a* = 0.011; *P<* 0.001) (no data available for Botryosphaeria dieback). For Chardonnay, on the contrary, the decline in wound susceptibility was faster for the Esca complex (*a* = 0.073) than for Botryosphaeria dieback (*a* = 0.023; *P* = 0.003) (**Supplementary Material S2**). Among the GTDs, the decline in wound susceptibility was faster for Botryosphaeria dieback with late-season pruning (*a* = 0.024) than with mid-season pruning (*a* = 0.01; *P* = 0.002) (**Table 5** and **Supplementary Material S2**).

When the estimates of *a* for main factors and interactions were compared with the estimate of *a* for the whole dataset (*a* = 0.019), significant differences were found as indicated in **Tables 2**–**5**.

## 4 Discussion

Since the 1970s, researchers have investigated the susceptibility of pruning wounds to GTD fungi as a function of DAP (number of days after pruning when the wounds were inoculated) and other factors. It is commonly accepted that the age of pruning wounds has a considerable effect on the susceptibility of wounds to GTD fungi, i.e., wound susceptibility decreases as the time increases between pruning and inoculation. The decline in susceptibility over time has been related to changes in environmental conditions affecting biological and physiological wound responses (Petzoldt et al., 1981; Eskalen et al., 2007; Úrbez-Torres and Gubler, 2011; Ayres et al., 2016). Many other factors affect wound susceptibility to infection, including grapevine variety, the pathogen that has been inoculated, pruning season, and other environmental conditions (Van Niekerk et al., 2011). As a consequence, the available information on the changes in wound susceptibility over time is inconsistent.

In this work, we conducted a quantitative analysis of the published data in order to synthesise the available information (Ferrer, 1998; Harrison, 2011; Ji et al., 2022). The studies analysed in this work included a wide variety of data: several grape varieties inoculated with different fungi, in different seasons, at different times after pruning, and in different countries around the world (including both the Northern and Southern Hemispheres). This variability well represents the complexity of the topic and highlights the need for synthesis. Because the studies included in our analysis involved artificial inoculation of wounds with fungi, disease incidence of wounds was probably higher than would occur with natural inoculation given that the inoculum dose is likely to be much higher with artificial than with natural inoculation (Elena and Luque, 2016). In support of this inference, pathogen recovery following artificial inoculation was lower with 10 ascospores per wound than with 100 or 1000 ascospores per wound (Elena et al., 2015; Ayres et al., 2022). The effect of inoculum dose and inoculation method used in different studies was not considered as a separate factor in this analysis because of the considerable variability among studies (see **Supplementary Material S3** for information on the inoculum of each study); therefore, possible effects of inoculum dose and inoculation method on parameter *a* estimation was not considered.

Our quantitative analysis showed a significant influence of the considered influencing factors on the infection incidence of GTD fungi inoculated at the time of pruning and on the rate of decline in infection incidence over time. Changes in wound susceptibility to fungal invasion after pruning followed a negative exponential distribution, a probability distribution that describes the time between events in a Poisson process, i.e., a process in which events occur continuously and independently at a constant average rate. In our case, the events consisted of the number of times wounds became infected over the total number of cases that wounds were inoculated at different times after pruning (i.e., disease incidence), and the constant average rate was the equation parameter *a* in equation [1]. We estimated *a* for the whole data set and for sub-sets referring to specific influencing factors (e.g., pruning period, identity of the GTD, etc.). In equation [1], *a* is related to the time required for the disease incidence to halve, so that the higher the estimate of *a*, the shorter the time required for disease incidence to decline from 1.0 to 0.5. To our knowledge, this is the first time an equation was fit to experimental data describing the temporal changes in wound susceptibility after pruning.

Equation [1] fit the whole dataset with R^2^ = 0.74; based on this equation, wounds remained susceptible to invasion by GTD fungi for months after pruning, and< 50% of the wounds remained susceptible at 6 weeks after pruning (**Figure 4**). We retrieved only one study in which data were collected for more than 5 months after pruning (Eskalen et al., 2007); in the latter study, no infection resulted from inoculations of 5-month-old or older pruning wounds.

The fit of equation [1] showed that the different factors (i.e., GTDs, pruning periods, grape varieties), and their interactions had a significant effect on the estimates of parameter *a*, so that the different curves showed a different pattern in the wound susceptibility over time. Therefore, the data dispersion around the fit of equation [1] for the whole dataset (**Figure 4**) was determined by specific factors that our quantitative analysis was able to identify. These factors are discussed in the following sections, as well as their possible implications for the practical management of GTDs.

Some data dispersion around the fitted line, however, remained unexplained by our quantitative analysis. Indeed, other factors may influence wound infection by GTD fungi and contribute to the imperfect fit of equation [1] to the observed data, which were not considered in our study because of a lack of information in the literature; they include the age of the wood that was pruned, the size of the cut surface, the extent of wound healing, the trellis system, the rootstock, plant vigor. Because most of the studies included in our analysis were conducted in vineyards, natural colonisation of pruning wounds by GTD fungi may have occurred in the period between two artificial inoculations. It follows that the incidence of GTD in the vineyard and weather conditions that may lead to infection and expression of disease symptoms, may also be a source of variability in the data that we used (Van Niekerk et al., 2011). Further studies aimed at clarifying the effect of all the above sources of variability in the response to fungal infection through pruning wounds should be carried out under controlled environmental conditions, in which the factor of interest can be evaluated by maintaining the others constant.

### 4.1 Effect of the GTDs

Our analysis showed that, at the time of pruning, disease incidence of wounds was higher for the fungi associated with Botryosphaeria dieback than for those associated with Esca complex or Eutypa dieback, which generally agrees with previous reports (Rolshausen et al., 2010; Amponsah et al., 2014; Ayres et al., 2016). In addition, pruning wounds remained susceptible to infection longer when inoculated with Botryosphaeria dieback fungi or Esca fungi than with Eutypa dieback fungi, except in the case of *Neofusicoccum luteum*, for which wound susceptibility decreased faster than for other Botryosphaeriaceae and *E. lata*. A shorter susceptibility of wounds to *E. lata* infection than to infection by other GTDs was observed by Ayres et al. (2016) and Herche (2006), but not by Elena and Luque (2016), who observed a similar decrease in wound susceptibility for Esca and Botryosphaeria dieback. Our result overall confirmed the potential threat of Botryosphaeriaceae species once they have established in a vineyard (Rolshausen et al., 2010) as well as the increasing prevalence of these species among the GTDs (Guerin-Dubrana et al., 2019).

An important limitation of our study is that no studies were found for the basidiomycetes involved in the EC. A possible reason is that these fungi have been isolated from infected vine trunks, but often with an incomplete understanding of their role in the disease aetiology (Surico et al., 2006; Bertsch et al., 2013; Gramaje et al., 2018). With the advent of metagenomics, reports on basidiomycetes are increasing (Del Frari et al., 2019; Bruez et al., 2020) as well as the studies on their role in symptom development (Cholet et al., 2021; Moretti et al., 2021; Pacetti et al., 2021); recently Brown et al. (2020) suggested that basidiomycetes may not require infection by *P. chlamydospora* in order to extensively colonize the wood. Studies on the susceptibility of pruning wounds to infection by basidiomycetes are then needed.

### 4.2 Effect of pruning season

Reports on the effect of pruning season on the susceptibility of pruning wounds to infection by GTD fungi are inconsistent. In California, USA, for instance, Petzoldt et al. (1981) found that pruning wounds made in December remained susceptible to infection by *E. lata* for a longer time than pruning wounds made in March. Similarly, Úrbez-Torres and Gubler (2011) showed that wounds were susceptible to *L. theobromae* and *N. parvum* for a longer time when pruning was done in early winter (with susceptibility lasting up to 84 days) than in early spring (with susceptibility lasting up to 12 days). Furthermore, Munkvold and Marois (1995) found that the percentage of wounds infected decreased faster with time when pruning was done in January or March than in previous months. Studies conducted in Spain, in contrast, documented a faster decrease in wound susceptibility when pruning was done in autumn compared to late pruning, but only when wounds were inoculated with *D. seriata* (Luque et al., 2014; Elena and Luque, 2016). Some studies reported that fresh pruning wounds are susceptible to infection regardless of the pruning season (Eskalen et al., 2007), and that adjustment of pruning season is ineffective in controlling wood diseases (Serra et al., 2008). Based on the differences among findings from experiments carried out under different conditions, Van Niekerk et al. (2011) concluded that the susceptibility of pruning wounds to infection may be less related to the time of year when pruning is done than to other factors, such as the disease investigated or more specifically the fungi implicated.

In our analysis, however, infection incidence for wounds inoculated at the time of pruning was overall lower when vines were pruned later in the season, and the reduction in the infection incidence over time after pruning for fungi causing Botryosphaeria dieback was faster when pruning was done in the late season than in earlier pruning periods. Úrbez-Torres et al. (2010) found that there was less spore dispersal by the Botryosphaeriaceae in the late dormant season than in winter, and this may contribute to the result of a lower incidence of wound infection with late versus early pruning.

We, therefore, confirm that the time of pruning is relevant for reducing infection of susceptible varieties and by fungi causing Botryosphaeria dieback, with late pruning being preferred, aware that seasonal variation might occur between regions caused mainly by climatic differences (Gramaje et al., 2018). Advantages of late pruning have been associated with “vine bleeding” in early spring, which can temporarily inhibit fungal penetration into the xylem vessels (Larignon and Dubos, 2000; Serra et al., 2008; Martínez-Diz et al., 2020) and can flush away fungal spores from the wound surface (Martínez-Diz et al., 2020). In addition, the low temperatures of winter may reduce the rate at which wounded tissues heal, increasing the time that they remain susceptible to infection (Petzoldt et al., 1981; Munkvold and Marois, 1995; Rolshausen et al., 2010). In contrast to our overall results, our analysis indicated that wounds of Thompson Seedless remained susceptible for a longer time when pruned later than earlier in the season (mid-season pruning), and this deserves further study.

### 4.3 Effect of the grape variety

In our quantitative analysis, differences were found among grape varieties for infection incidence of wounds inoculated at pruning (t_0_) and after pruning. For the t_0_ data, however, data variability and unbalanced replications caused some under-dispersion in the GLM model (Sellers and Morris, 2017). Additional data are needed to determine whether the degree to which the probability of a pruning wound becoming infected is influenced by grape variety, and to which extent.

Our analysis showed that pruning wounds remained susceptible to GTDs for a longer time on Sauvignon Blanc than on other varieties. Sauvignon Blanc was previously reported to be especially susceptible to GTDs (Borgo et al., 2008; Bertsch et al., 2013; Murolo and Romanazzi, 2014; Sosnowski et al., 2022). Similarly, wounds of Cabernet Sauvignon remained susceptible to infection for a longer time than wounds of other red varieties, such as Grenache, Pinot noir, Merlot, or Shiraz. Differences in the susceptibility of grapevines to GTDs may be related to a variety of factors such as differences in sensitivity to fungal toxins, the rate of toxin degradation in wood tissue, and the time required for recognition of the fungal invasion and consequent activation of defense mechanisms (Wagschal et al., 2008), which involve stilbenes, other polyphenols, and proteins involved in primary metabolism (Borgo et al., 2016; Fontaine et al., 2016a; Fontaine et al., 2016b). Xylem morphology, and especially xylem vessel size may also contribute to differences in susceptibility among varieties (Pouzoulet et al., 2014; Fischer and Peighami Ashnaei, 2019; Claverie et al., 2020). It is also important to notice that a higher number of data was available for Cabernet Sauvignon than for Grenache, Pinot noir, Merlot and Shiraz so that our estimation of parameter *a* would be more accurate for Cabernet Sauvignon than for other varieties. Additional work is then needed to add robustness to our interpretations.

## 5 Limitations

Following the general objectives of systematic literature search and quantitative analysis of literature data, our work makes a synthesis of published information and projects some relevant aspects that need further research. The number of studies we found and used is limited and most of them did not include complete information on the experimental variability, thus not permitting the calculation of the effect sizes and the execution of a formal meta-analysis. Unfortunately, this limitation has been frequently faced by other authors (Koricheva and Gurevitch, 2014; Nakagawa et al., 2017). Meta-analysis is used to combine common effect sizes across studies and accounts for statistical non-independence, which occurs when data points (or effect sizes) are related to each other (for example when multiple points or effect sizes come from a single study). Failing to account for non-independence is generally considered a limitation that can lead to a loss of information or erroneous conclusions (Nakagawa et al., 2017). Even though the execution of a formal meta-analysis would lead to more robust conclusions about the susceptibility of pruning wounds to infection by GTD fungi, our manuscript represents a useful assessment of the information published so far.

Our work also suffers from the incomplete knowledge about GTDs we discussed before. Systematic literature review, however, does not have the ambition of elucidating all the aspects related to a topic, but it is useful for identifying knowledge gaps and orientating further research. Complex interactions between cultivar, biogeography, wood healing, the wood fungal pathobiome, pruning time, and environmental conditions, need further investigations.

Furthermore, our work does not claim that pruning wounds are the sole or most important infection pathway for GTD fungi. Other wood injuries caused by frost, hail, and mechanical harvesting can also act as points of entry. Soil is also considered a possible reservoir of inoculum, and roots an entry point for *Phaeoacremonium* spp., *Pa. chlamydospore* (Whiteman et al., 2002; Whiteman et al., 2005; Agustì-Brisach et al., 2013), and *D. seriata* (Whitelaw-Weckert et al., 2006; Whitelaw-Weckert et al., 2013), but for other Botryosphaeriaceae has not been observed (Amponsah et al., 2012a; Billones-Baaijens et al., 2013b; Billones-Baaijens and Savocchia, 2019). Soil is also a source of inoculum for black-foot pathogens (Halleen et al., 2006; Agustí-Brisach and Armengol, 2013; Agustì-Brisach et al., 2013; Agustí-Brisach et al., 2014) but as most GTD fungi utilize air-borne inoculum, its role as a reservoir of inoculum needs to be further investigated (Billones-Baaijens and Savocchia, 2019; Claverie et al., 2020).

## Data availability statement

The original contributions presented in the study are included in the article/**Supplementary Material**. Further inquiries can be directed to the corresponding author.

## Author contributions

MCR was the principal investigator. MCR and SEL extracted the data. MCR and VR conducted the statistical analysis and drafted the manuscript. VR, IS and SEL edited and revised the manuscript. All authors contributed to the article and approved the submitted version.
